# The Absence of the Verumontanum at Voiding Cystourethrography as a Sign of Prostate Maldevelopment

**DOI:** 10.1155/2011/982709

**Published:** 2010-11-30

**Authors:** A. L. Valentini, P. Ferrara, C. Manzoni, A. P. Mancini, S. Pulitanò, L. Bonomo

**Affiliations:** ^1^Department of Bioimaging and Radiological Sciences, Institute of Radiology, A. Gemelli Hospital, Catholic University of Rome, Largo A. Gemelli 8, 00168 Roma, Italy; ^2^Department of Pediatrics, A. Gemelli Hospital, Catholic University of Rome, 00168 Rome, Italy; ^3^Department of Pediatric Surgery, A. Gemelli Hospital, Catholic University of Rome, 00168 Rome, Italy; ^4^Department of Bioimaging and Radiological Sciences, Campus Bio-Medico University, 00100 Rome, Italy

## Abstract

Prostate maldevelopment in prune-belly syndrome has only been described at necropsy. No reports are available in the “in vivo” studies. The absence of the verumontanum at voiding cystourethrography correlates with verumontanum and prostate hypoplasia. This radiographic sign can represent the earliest finding in prostate maldevelopment and might contribute to the “in vivo” assessment of the disease, especially in doubtful cases.

## 1. Introduction

The prune-belly syndrome (PBS) is a rare condition, with a ratio of 1 to 40.000, occuring almost exclusively in males, and it is classically characterized by the deficit of the anterior abdominal muscular wall, bilateral cryptorchidism, and genitourinary alterations. Bilateral cryptorchidism might not always be observed, and urinary tract alterations might be monolateral or completely absent, but the deficit of the abdominal musculature, generalized or localized to one of the abdominal quadrants, is always present [1, 2]. Ureters are dilated and tortuous especially in the lower tract; this is due to an increased connective and reduced muscular components of the ureteral wall. The posterior urethra is also large, and an enlarged and thickened bladder is usually present. Renal dysplasia can be associated, while prostate hypoplasia is only reported in the necroscopy studies [3–5]. Orthopedic, gastrointestinal, respiratory, and cardiac problems might also be found. In our department, in the suspect of PBS diagnosis an abdominal ultrasound (US) and voiding cystourethrography (VCUG) under antibiotic prophylaxis are usually performed early in life to evaluate the kidney and bladder morphology, to confirm undescended testis, and to exclude vesicoureteral reflux (VUR) or urethral obstruction. Special attention should be given to the verumontanum (VM) at VCUG voiding phase, because the absence of this sign could represent the earliest finding in prostate maldevelopment. The clinical history and instrumental examinations of a 10-year-old male followed at our department since birth are reported, to support the hypothesis that the VM absence at VCUG can help diagnose PBS when external features are not typical and/or the clinical diagnosis of PBS is doubtful.

## 2. Case Report

**Figure 1:**
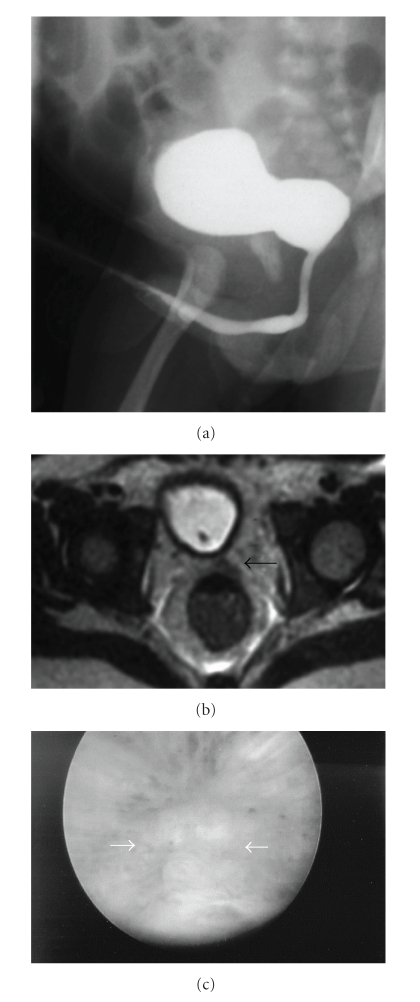
Voiding cystourethrography, magnetic resonance imaging, and urethroscopy in prune-belly syndrome. (a) In a 2-month-old child, the voiding phase of cystourethrography shows a large bladder neck without VM salience in the posterior urethra. (b) In the same patient at the age of 10, T2-weighted axial scan shows the absence of the right deferent and seminal vesicle, while the left seminal vesicle is tubular (black arrow) and the prostate gland is not detected. (c) Urethroscopy confirms the verumontanum hypoplasia, which is quite appreciable (arrows).

PBS was first hypothesized when the child was about 2 months old on the basis of bilateral cryptorchidism, severe nonfunctioning right kidney dysplasia, bilateral ureteral dilation, and thickened bladder. However, the posterior urethra was nonobstructed; as also described in PBS cases, the deficit of the abdominal musculature was quite appreciable and the belly classic aspect was not consistently shown. At this time, an unusual absence of the normal VM image at VCUG was noted (Figure 1(a)) while no information on the prostate gland was reported at the US evaluation.

When the patient was 2 years old, the nonfunctioning dysplastic right kidney and the left testis were removed. In the following eight years, all clinical and instrumental examinations (renal angioscintigraphy, US, and VCUG), periodically performed, showed a satisfactory control of the renal insufficiency but a persistent left ureteral dilation, without any obstruction, and an inconstant left VUR. The VM image, usually well visible in children, was never shown at further VCUG controls. At the age of 10, the patient was hospitalized with a severe urinary tract infection. Cultures of both urine and blood were positive for Escherichia coli. At this time, magnetic resonance (MR) which was planned to confirm the US diagnosis of renal abscess also documented a very thickened urethral wall and the absence of a normally developed prostate gland. The absence of the right deferent and seminal vesicle was associated (Figure 1(b)). The urethroscopy stated VM hypoplasia (Figure 1(c)).

## 3. Discussion and Conclusion

The PBS diagnosis is usually based on the deficit of the anterior abdominal muscular wall, bilateral cryptorchidism, and genitourinary alterations. In previously reported PBS cases, prostate and VM hypoplasia have been well documented at necropsy [3–5], but not in “in vivo” studies. This might be related to the different degrees of anatomical abnormalities, to the presence of complete or incomplete forms of PBS, or to pseudo-PBS cases (uropathy, cryptorchidism, but normal abdominal wall) in which the prostate gland maturation is slightly higher especially in stromal elements [4, 6]. However, other causes should be considered. The prostate gland of normal males has an immediate after birth regression, growth until puberty, and maturation between 14 and 18 years. In normal children aged between 7 months and 13 years, the normal prostate development and the normal growth until puberty have been investigated by US for the prostate size [7], with volume ranging between 0.4 and 5.2 cc. In PBS, the presence of a thickened bladder wall and relaxation of the abdominal musculature might not permit an adequate investigation by US in children. Therefore, prostate hypoplasia might not be correctly evaluated, as was in our case. MR is one of the most accurate techniques allowing a satisfactory evaluation of the prostate gland, because the quality of the examination is not influenced by the altered abdominal wall and bladder anatomy (thickened wall and loss of trabecular aspect). However, MR requires anesthesia or deep sedation, especially in small children. Moreover, the diagnosis of prostate maldevelopment can be missed, because of the very small prostate size around birth in healthy children too [7]. The absence of VM at VCUG, especially in a technically satisfactory study, can suggest the presence of VM hypoplasia based on a VM too small to be radiologically visible. The association between VM being either small or absent and prostate hypoplasia is well known [3], and it is easily explained by the embryological development of VM which results from the epithelial proliferation between the utricle cord and the urogenital sinus; these later both contribute to the prostatic stroma and epithelium development [4, 8]. The absence of the VM at VCUG correlates with VM hypoplasia and therefore with prostate maldevelopment. Our case was not typical for PBS, since the deficit of anterior abdominal wall was extremely mild and the urethra was not obstructed [3]. The absence of the VM at VCUG early suggested the diagnosis of prostate maldevelopment that was confirmed at the late MR and helped us in the assessment of the patient's disease. In the author's opinion, this simple radiographic sign should be stressed as an early “sentinel sign” for prostate maldevelopment and might contribute to the “in vivo” assessment of PBS cases, especially when the typical external features are mild or incomplete or the diagnosis is doubtful.
